# Burning Mouth Syndrome Following Covid Vaccination: A Case Report

**DOI:** 10.1002/ccr3.70329

**Published:** 2025-04-29

**Authors:** Mustafa Broachwala, Dylan W. Banks, David S. Jevotovsky, Whitman Oehlermarx, Shravani Durbhakula

**Affiliations:** ^1^ Department of Anesthesiology and Pain University of California San Diego San Diego California USA; ^2^ Department of Physical Medicine and Rehabilitation New York University New York New York USA; ^3^ Department of Anesthesiology Vanderbilt University Medical Center Nashville Tennessee USA

**Keywords:** acupuncture, burning mouth syndrome, COVID‐19 vaccines, xerostomia

## Abstract

This is the first reported case of burning mouth syndrome resulting from the Pfizer‐BioNTech COVID‐19 vaccine. It highlights the efficacy of a unique multimodal treatment regimen, including zinc supplementation, acupuncture, and topical capsaicin.

## 1 Introduction

Burning Mouth Syndrome (BMS) is an idiopathic disorder defined as a burning sensation most often affecting the tongue, hard palate, and/or lips. This painful condition occurs in patients with normal oral mucosa in whom a medical or dental cause has been excluded [1, 2]. Diagnostic criteria include intraoral burning or pain for > 2 h per day for > 3 months [3]. The onset usually is spontaneous but can occur with physical or psychological trauma [4]. Furthermore, there may be associated xerostomia or dysesthesia. Only about 4% of cases resolve over a 5‐year period without treatment, with 28% reporting moderate improvement with or without treatment [5, 6]. Reports of prevalence fluctuate from 1% [7] to 15% [8], likely due to it being a challenging diagnosis with different diagnostic criteria between North American and European populations [4]. It is more common in women than men (33:1), especially those who are peri‐ or post‐menopausal [9]. BMS is often comorbid with other chronic pain disorders [10]. The etiology and pathophysiology are unclear, though central, peripheral, and psychological factors play a role [3, 11].

COVID‐19 vaccination efforts have been revolutionary in mitigating a global pandemic. The Pfizer‐BioNTech COVID‐19 vaccine is an mRNA vaccine that induces the formation of viral spike protein to invoke immunity against SARS‐CoV‐2 infection. The most common systemic side effects include headaches, fevers, chills, and myalgias [12]. In addition, although rare, some case reports have demonstrated neurological manifestations, including Guillain–Barré syndrome (GBS) [13], transverse myelitis [14], and facial nerve palsy [15].

Regarding oral manifestations following COVID‐19 vaccination, one preliminary survey of 223 participants showed a 2.7% occurrence of burning sensations [16]. However, a formal diagnosis of BMS was not established in this survey. In addition, a case series by Chun et al. [17] reported oral lesions and inflammation. This same case series showed a BMS case in a patient receiving the vector‐based Astrazeneca vaccine and an exacerbation of a burning sensation in a patient formerly diagnosed with BMS [17]. A narrative review of oral manifestations after either COVID‐19 infection or vaccine reported only 1 case of chronic oral burning after an unspecified Covid vaccine, in which the patient was ultimately diagnosed with Sjögren's disease [18].

In the below report, we present the first known case of a male patient who presented with burning mouth syndrome subsequently after Pfizer COVID‐19 vaccination, as well as a unique, multimodal approach to effective treatment.

## 2 Case History/Examination

A 39‐year‐old male with a past medical history of hypoplastic right lung, bipolar disorder (on lithium and oxcarbazepine), attention deficit hyperactive disorder, and migraines presented to the clinic with a burning sensation located on the dorsal aspect of his tongue with an altered sensation of old food residues and xerostomia. This sensation was constant and fluctuated in severity from a 6 to 10.

One year earlier, the patient received the first Pfizer COVID‐19 vaccination. He noted that about 90 min after the vaccination, he began to feel a burning sensation in his feet that extended up towards his mouth and cheeks. He had an episode of presumed vasovagal syncope shortly after that resolved without treatment or further workup. The burning sensation in his mouth has persisted since. He also noted that after the second vaccination dose 1 month after the first, he developed stiffness throughout his body and could not eat for 2 days. Although the burning pain in his mouth had slightly improved since the initial onset, it still affected his ability to work.

The patient subsequently underwent an MRI of the head, which was unremarkable. He also underwent a biopsy of the tongue, which did not show any pathology.

Prior to presenting in our clinic, the patient tried gabapentin, Lyrica, dexamethasone oral wash, saliva supplements, and CBD with titration to maximum dosages without relief. He noted that oral lidocaine and nystatin swish and spit provided mild relief for 30 min at a time.

The physical exam was unremarkable, including a normal neurologic examination as well as normal‐appearing oral mucosa without lesions or erythema.

We prescribed the patient oral capsaicin 0.02% washes 4 times a day, which provided transient relief for 1 h at a time. We also recommended increasing his amitriptyline from 50 to 75 mg BID and supplementation with alpha lipoic acid. Neither of these provided any relief. Lastly, daily oral zinc supplementation was recommended, and the patient noted that this reduced his pain significantly.

At his 2‐month follow‐up appointment post zinc initiation, the patient reported that he started acupuncture on the tongue. This combined with zinc and oral capsaicin washes provided a near‐complete resolution of his pain and xerostomia.

## 3 Differential Diagnosis

In the absence of any alternative medical explanation—including infection (e.g., herpes zoster, candidiasis), Sjögren's syndrome, or diabetes—and in the setting of normal oral mucosa confirmed on biopsy, a diagnosis of BMS was made.

## 4 Conclusion and Results

In this case, the patient presented with a history and physical exam consistent with a diagnosis of BMS. The patient gained significant benefits from oral capsaicin, oral zinc supplementation, as well as acupuncture of the tongue. This is the first known case in which a unique, multimodal approach to treatment was effective in a patient with new‐onset BMS immediately following the first dose of the Pfizer COVID‐19 vaccination.

## 5 Discussion

### 5.1 BMS Pathophysiology and Hypothesized Mechanisms

Although the pathophysiology of BMS is unclear, several mechanisms have been proposed, including peripheral, central, and autonomic factors. Peripherally, the lingual mucosa has been shown to have fewer small‐diameter nerve fibers, as well as upregulation of the TRPV1 receptor, which is sensitive to heat and selective for capsaicin [19]. One study also demonstrated abnormalities in nerve conduction via electromyography [20]. Centrally, there may be a decrease in GABA‐mediated pain inhibition in the dorsal horn of the spinal cord, specifically inhibiting the glutamate/NMDA‐mediated central sensitization [21]. Furthermore, it has been proposed that a downregulation of the nigrostriatal dopaminergic‐mediated pain inhibition may be involved [22].

It is possible that there is some sympathetic mediation in BMS given a case report demonstrating successful treatment of BMS with sphenopalatine ganglion (SPG) block [23]. Innervation of the anterior tongue is provided in part by the lingual nerve, which is a branch of the mandibular branch (V3) of the trigeminal nerve. These same nerves go on to synapse in the SPG. Increased sympathetic outflow may be further implicated through a Doppler flowmetry study that demonstrated a reduction in oral blood flow among BMS patients [24]. While further research needs to be done in this regard, in future cases refractory to multimodal, noninterventional treatments, SPG block and other treatments toward sympathetic mediation should be further investigated.

### 5.2 Pfizer Vaccine‐Induced BMS Pathophysiology and Hypothesized Mechanisms

Neurological manifestations of COVID‐19 vaccines are rare but have been noted in several case reports. A recent review by Sriwastava et al. in 2022 showed mRNA vaccines (Pfizer and Moderna) to have fewer neurologic manifestation cases than vector‐based vaccines (Astrazeneca) [25]. This review linked 9 cases of neurological manifestations to Pfizer vaccination. The reported events included acute encephalomyelitis (1 case) [26], Guillain‐Barré Syndrome (3 cases) [27, 28, 29], Bell's Palsy (3 cases) [15, 30, 31], small fiber neuropathy (1 case) [27], and phantosmia (1 case) [32]. In most incidences listed above, symptoms manifested after the second dose. Only two cases had symptom onset after the first dose, and these manifested in Bell's Palsy—one and 5 days after administration.

Based on existing research, it appears that CNS‐related adverse events related to Pfizer vaccination are rare and may likely be coincidental [25]. This would lead us to believe that the CNS is not primarily implicated in the mechanism of vaccine‐induced BMS.

One mechanistic hypothesis is that autoimmune processes mediate post‐vaccine peripheral neurological events. The mRNA vaccines, including Pfizer, introduce mRNA into cells and instruct the cells to identify the spike protein found on the surface of SARS‐COV‐2. Subsequently, our bodies recognize the spike protein as an invader and produce antibodies against it. Later, these antibodies are able to recognize the virus before it can cause illness. In some patients, this can trigger an autoimmune process leading to the production of antibodies against the myelin and subsequent peripheral nervous system disease. A similar theory is the potential cross‐reaction between spike protein antibodies and sialic acid, considering that the spike protein can bind to cell surface sialic acids, including those on the ACE‐2 receptor [33]. Based on the Sriwastava et al. review, GBS is one of the more common neurologic manifestations following mRNA vaccination, and it is a disease known to have a strong autoimmune component [25]. Idiopathic autoimmunity has been postulated in other manifestations discussed above, such as Bell's Palsy and small fiber neuropathy [15, 27, 30, 31]. Therefore, autoimmune pathophysiology cannot be ruled out in the case of Pfizer vaccine‐induced BMS.

### 5.3 BMS Treatment Regimen

This case demonstrated the successful management of post‐vaccine BMS through a unique multimodal approach. Oral capsaicin 0.02% rinse provided the patient with good, transient relief. Capsaicin inhibits the biosynthesis and axonal transport of substance P in afferent sensory nerve fibers, leading to decreased nociception. One smaller study demonstrated similar, short‐lasting results with oral capsaicin 0.02% rinse vs. placebo in patients with BMS [34]. Unfortunately, the short‐duration effect and burning sensation are limiting and may be paradoxical in treatment.

Oral daily zinc supplementation also provided the patient with significant relief of the pain symptoms. Although we did not check zinc levels in this case, decreased zinc levels have been reported among patients with BMS [35, 36, 37]. One study of 89 patients found that zinc supplementation, vitamin B complex, with or without topical capsaicin, provided significant reduction in pain and burning in BMS [38]. Another study demonstrated the superior benefit of zinc supplementation compared to oral steroid rinse in those who are zinc deficient at a 6‐month follow‐up [36]. Overall, further research is warranted regarding the utility of zinc supplementation for use in BMS, but it may be considered an effective, safe, and low‐cost treatment modality.

Nearly 2 months of daily acupuncture was beneficial in not only this patient's pain but xerostomia symptoms as well. There are several studies that show a significant reduction in pain and burning sensation after acupuncture, although the sample size for these studies was smaller [6, 39, 40, 41]. One of these studies demonstrated no significant difference between acupuncture and clonazepam, except that the acupuncture group did not have worsening Montreal Cognitive Assessment (MoCA) scores, demonstrating a favorable side effect profile as a treatment modality [42]. Although the mechanism of action for its analgesic effect is not well understood and likely multifactorial, Scardina et al. demonstrated improved oral microvasculature in BMS patients via capillaroscopy, which may infer improved washout of inflammatory products and subsequent remission of pain [39]. Beyond pain, the literature regarding acupuncture's therapeutic effect on xerostomia remains insufficient. One review of 10 randomized control trials suggested there may be an improvement in xerostomia [43]. More extensive studies are required to determine the true efficacy of acupuncture, but the current literature seems promising and offers a safe alternative or adjuvant treatment modality.

In conclusion, although neurologic manifestations following vaccination are rare, it is important for physicians to be aware of such cases and promote further research in this regard. Furthermore, further research is warranted regarding nonconventional treatment modalities for BMS to provide comprehensive guidelines for this rare disease.

## Author Contributions

**Mustafa Broachwala:** conceptualization, investigation, resources, supervision, validation, writing – original draft, writing – review and editing. **Dylan W. Banks:** investigation, resources, validation, writing – review and editing. **David S. Jevotovsky:** supervision, writing – review and editing. **Whitman Oehlermarx:** writing – review and editing. **Shravani Durbhakula:** conceptualization, supervision, validation, writing – original draft, writing – review and editing.

## Ethics Statement

This report did not require review by the ethics committee at our respective institutions.

## Consent

Written consent was obtained for the publication of this case.

## Conflicts of Interest

The authors declare no conflicts of interest.

## Data Availability

The authors have nothing to report.
